# Role of the Preoptic Area in Sleep and Thermoregulation

**DOI:** 10.3389/fnins.2021.664781

**Published:** 2021-07-01

**Authors:** Rebecca Rothhaas, Shinjae Chung

**Affiliations:** Department of Neuroscience, Perelman School of Medicine, Chronobiology and Sleep Institute, University of Pennsylvania, Philadelphia, PA, United States

**Keywords:** sleep, thermoregulation, body temperature, preoptic area of the hypothalamus, neural circuits

## Abstract

Sleep and body temperature are tightly interconnected in mammals: warming up our body helps to fall asleep and the body temperature in turn drops while falling asleep. The preoptic area of the hypothalamus (POA) serves as an essential brain region to coordinate sleep and body temperature. Understanding how these two behaviors are controlled within the POA requires the molecular identification of the involved circuits and mapping their local and brain-wide connectivity. Here, we review our current understanding of how sleep and body temperature are regulated with a focus on recently discovered sleep- and thermo-regulatory POA neurons. We further discuss unresolved key questions including the anatomical and functional overlap of sleep- and thermo-regulatory neurons, their pathways and the role of various signaling molecules. We suggest that analysis of genetically defined circuits will provide novel insights into the mechanisms underlying the coordinated regulation of sleep and body temperature in health and disease.

## Introduction

In homeothermic animals, sleep, and body temperature are tightly interconnected. The ambient and body temperature influence the amount of sleep. Warming up the body such as taking a warm bath or passive heating promotes sleep (Horne and Staff, 1983; Horne and Shackell, 1987; Bunnell et al., 1988; Morairty et al., 1993; Kräuchi et al., 1999). Animals show preparatory behaviors before sleep such as nest building, social huddling, seeking warm places and curling up to reduce body heat loss (Gordon et al., 2014; Harding et al., 2018; Peever, 2018). Human studies showed that core body temperature starts to decrease a few hours before the sleep onset (Campbell and Broughton, 1994; Landolt et al., 1995; Krauchi and Deboer, 2010). The brain and core temperature in rats also start to decrease before sleep onset, a phenomenon observed for different ambient temperatures (ranging from 10°C to 24.6°C) (Alföldi et al., 1990; Franken et al., 1992a, b; Campbell and Broughton, 1994; Landolt et al., 1995). During non-rapid eye movement (NREM) sleep, the brain, and core temperature then decrease with similar magnitudes irrespective of the ambient temperature. In contrast, the tail temperature significantly rises at the NREM sleep onset (at 10 and 21°C) indicating increased vasodilation. NREM sleep is a state with a low level of energy metabolism, cardiovascular, and thermoregulatory functions to conserve energy while feeding is reduced. Central autonomic nervous system activity regulating cardiovascular function and breathing as well as endocrine function is set to support this need during NREM sleep. Energy conservation and cooling of the body and brain are thought to be major functions of the tight interconnection of sleep and thermoregulation (Berger, 1984; McGinty and Szymusiak, 1990; Heller and Ruby, 2004).

The preoptic area of the hypothalamus (POA), located in the anterior part of the hypothalamus, is known to be critical for both thermoregulation and sleep. In addition, it is also involved in regulating energy homeostasis, parenting, and sexual behaviors, each of which is controlled by dedicated circuits (Simerly, 2002; Dulac et al., 2014; McKinley et al., 2015; Kohl and Dulac, 2018; Yu et al., 2018; Tsukahara and Morishita, 2020). However, how these circuits are interconnected within the POA and beyond to coordinate these complex behaviors is still not very well understood.

**FIGURE 1:**
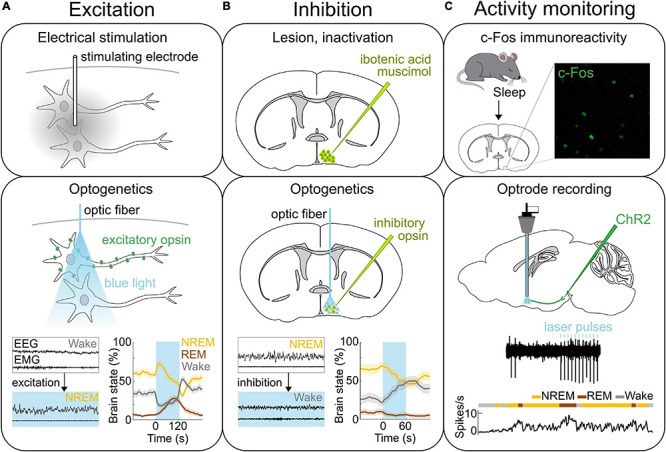
Methods of neural activity manipulation and monitoring. **(A)** Methods for activating neurons. Top, Electrical stimulation allows for activating neurons in the area where the stimulating electrode is located. Middle, Expressing light-activated excitatory opsins such as channelrhodopsin-2 (ChR2) in genetically-defined neurons allows for the manipulation of specific neural populations on the order of milliseconds using light (Deisseroth, 2015; Grosenick et al., 2015; Deisseroth and Hegemann, 2017; Kim et al., 2017). Bottom, Optogenetic stimulation of ChR2 expressing GABA^POA→TMN^ neurons promotes sleep. Bottom left, Example EEG and EMG traces before and during laser stimulation (blue shading). Bottom right, Percentage of time in NREM, REM or wake state before, during, and after laser stimulation (blue shading). **(B)** Methods for inactivating neural activity. Top, The specific brain region can be lesioned or inactivated for example by injecting the neurotoxin ibotenic acid or muscimol. Middle, Expression of light sensitive inhibitory opsins in genetically-defined neurons enables the inhibition of specific neural populations (Wiegert et al., 2017). Bottom, Optogenetic inhibition of the iC++ expressing GABA^POA→TMN^ neurons suppresses sleep. Left, Example EEG and EMG traces before and during laser stimulation (blue shading). Right, Percentage of time in NREM, REM or wake state before, during, and after laser stimulation (blue shading). **(C)** Methods for recording neural activity. Top, c-Fos immunohistochemistry has been used to detect neurons in the POA that are activated following sleep. Example POA neurons stained for expression of c-Fos (green) following deprivation-induced sleep rebound. Middle and bottom, Optrodes allow for identifying ChR2-expressing neurons and recording their spiking activity during sleep and wakefulness. An example recording from GABA^POA→TMN^ neurons. Reproduced from Chung et al. (2017). Sagittal and coronal brain scheme adapted from Allen Mouse Brain Atlas (©Allen Brain Atlas API. Available from http://api.brain-map.org).

The recent development of neuroscience tools for circuit analysis has significantly helped to identify the specific cell types and regions within the POA that are crucial for sleep and body temperature regulation (Figure 1). We will review our current knowledge on the neural mechanisms underlying sleep and thermoregulation. Finally, we will discuss key questions that remain unresolved to elucidate how sleep and body temperature are coordinated within the POA.

## Role of the POA in Sleep Regulation

In the 1920s, lesions in the POA were found to be associated with insomnia in human patients affected by encephalitis lethargica (von Economo, 1930). In rats, various brain regions were systemically lesioned, and the POA was confirmed to be a critical sleep center (Nauta, 1946). This finding was also reproduced in cats showing that the size and location of the lesions within the POA are correlated with the severity of sleeplessness (McGinty and Sterman, 1968). Conversely, electrically stimulating the POA in cats promotes sleep (Sterman and Clemente, 1962b; Figure 1A). Furthermore, insomnia induced by POA lesions after injection of the neurotoxin ibotenic acid (Sallanon et al., 1989; Lu et al., 2000; Figure 1B) was reversed by injection of muscimol into the ventrolateral part of the posterior hypothalamus (Sallanon et al., 1989) suggesting that the sleep-promoting effect of POA neurons is mediated by their inhibitory projections to the posterior hypothalamus. c-Fos immunohistochemistry revealed the existence of sleep-active neurons in the POA (Sherin et al., 1996; Gong et al., 2004; Gvilia et al., 2006; Chung et al., 2017; Figure 1C). In sleeping rats, the number of c-Fos labeled neurons is elevated in the ventrolateral preoptic area (VLPO) and median preoptic nucleus (MnPO) and is correlated with the amount of sleep before sacrificing the animal (Sherin et al., 1996; Gong et al., 2004). Electrophysiological recordings in the POA of rats and mice further demonstrated *in vivo* the existence of neurons that are active during rapid eye movement (REM) and/or NREM sleep (Koyama and Hayaishi, 1994; Osaka and Matsumura, 1994; Szymusiak et al., 1998; Takahashi et al., 2009). The firing rate of VLPO neurons was elevated during the homeostatic sleep rebound following deprivation, but remained at low levels throughout the deprivation period (Szymusiak et al., 1998). Similarly, c-Fos levels in VLPO neurons were not elevated, if rats were sacrificed right after sleep deprivation (Sherin et al., 1996). These results suggest that the activity of VLPO neurons reflects the amount or intensity of sleep, rather than the sleep propensity accumulated during wakefulness. TetTagging was applied to express the excitatory hM3DGq receptor in c-Fos activated neurons followed by a deprivation-induced sleep rebound (Zhang et al., 2015). Chemogenetically reactivating these neurons enhanced sleep confirming their causal role in promoting sleep. This study further suggests that sleep-promoting neurons are located throughout a broader area within the POA than previously thought.

c-Fos immunohistochemistry also provided evidence for a role of the POA in REM sleep regulation. c-Fos expression was examined in the VLPO and MnPO during spontaneous sleep, REM sleep restriction and subsequent REM sleep recovery (Gvilia et al., 2006). The number of c-Fos neurons was highest in the REM sleep restriction group suggesting that they are involved in the homeostatic regulation of REM sleep. Furthermore, when REM sleep was enriched after periods of dark exposure, the number of c-Fos-expressing cells in the extended VLPO (the area medially and dorsally extending from the VLPO) was correlated with the amount of REM sleep and its lesion decreased REM sleep (Lu et al., 2002).

### Molecular and Circuit Level Characterization of POA Sleep Neurons

A high percentage of sleep-active neurons identified using c-Fos immunohistochemistry has been shown to express galanin in multiple mammalian species including rats, mice, degus, and cats (Sherin et al., 1998; Gaus et al., 2002). In rats, galanin labels a pure population of c-Fos-labeled sleep neurons in the VLPO, whereas in mice about 33% of c-Fos-labeled wake-active neurons also contain galanin (Gaus et al., 2002), indicating that this neuropeptide labels diverse neural populations in mice. Optogenetic stimulation of galanin neurons promotes wakefulness (Chung et al., 2017) or NREM sleep (Kroeger et al., 2018) depending on the stimulation frequency. Chemogenetic stimulation of galanin neurons promoted sleep (Kroeger et al., 2018). Surprisingly, chronic ablation of these neurons through cell type specific expression of the apoptotic Caspase3 increased the amount of NREM sleep, while reducing wakefulness (Ma et al., 2019). The duration of NREM sleep and wake bouts was significantly decreased, suggesting that sleep is highly fragmented in the absence of galanin neurons. Ablating galanin neurons also impaired sleep homeostasis: mice slept less and exhibited a reduced increase in delta power during NREM sleep following sleep deprivation. Using fiber photometry, a recent study recorded the calcium activity of the galanin population in the POA and found that they are most active during REM sleep [published in preprint form Miracca et al. (2020)]. Future studies to measure the activity of galanin neurons at the single cell level will further clarify their role in sleep control.

**FIGURE 2:**
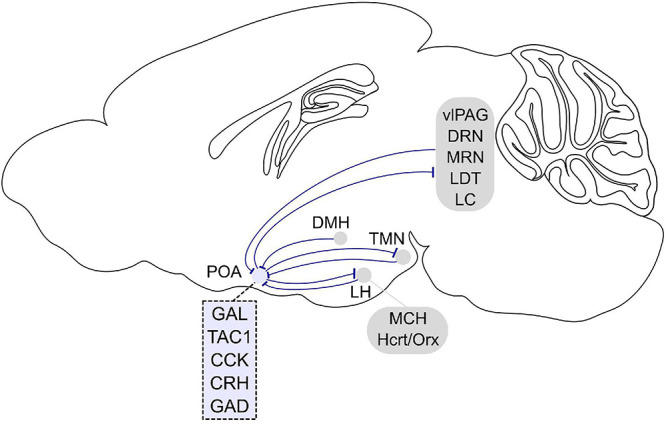
Afferent and efferent projections of sleep regulatory POA neurons. Sleep promoting GABAergic POA neurons inhibit histamine neurons in the tuberomammillary nucleus (TMN). In turn, histaminergic TMN neurons project to the POA, and histamine indirectly inhibits putative VLPO sleep neurons. POA GABAergic neurons also densely project to the lateral hypothalamus (LH) and directly inhibit hypocretin/orexin (Hcrt/Orx) neurons. The POA in turn receives inputs from the LH, and a small fraction of neurons contain melanin-concentrating hormone (MCH) or Hcrt/Orx. GABAergic neurons in the LH also directly innervate POA galanin neurons. The POA receives inputs from dorsomedial hypothalamus (DMH) galanin neurons that are NREM promoting and REM suppressing. POA neurons project to brainstem regions such as the ventrolateral periaqueductal gray (vlPAG), raphe nuclei (DRN, MRN), laterodorsal tegmental nucleus (LDT), and locus coeruleus (LC). In turn, the POA receives inputs from these brain stem regions. Molecular markers labeling sleep regulatory POA neurons are galanin (GAL), tachykinin 1 (TAC1), cholecystokinin (CCK), and corticotropin-releasing hormone (CRH). Most sleep neurons are GABAergic and express GAD (glutamic acid decarboxylase). Sagittal brain scheme adapted from Allen Mouse Brain Atlas (©Allen Brain Atlas API. Available from http://api.brain-map.org).

A recent study used cell type and projection specific targeting and gene profiling to identify molecular markers that are enriched in the POA neurons that promote sleep and are sleep-active (Chung et al., 2017). Such sleep neurons are thought to promote sleep by suppressing arousal systems in downstream areas including the tuberomammillary nucleus (TMN), raphe nucleus, and locus coeruleus (LC) (Saper and Fuller, 2017; Scammell et al., 2017). Sleep-activated c-Fos neurons project to the TMN, a brain region containing wake-active histamine neurons (Sherin et al., 1996; Chung et al., 2017; Figure 2). Monosynaptic rabies tracing revealed that POA neurons that directly innervate histamine neurons in the TMN are largely GABAergic (γ-aminobutyric-acid-releasing) and express c-Fos in response to sleep (Chung et al., 2017; Saito et al., 2018). GABA release in the posterior hypothalamus of cats increases during NREM sleep compared to wakefulness, likely to facilitate NREM sleep and one of the potential sources of GABA could be the POA (Nitz and Siegel, 1996). To investigate the causal role of POA GABAergic neurons projecting to the TMN (GABA^POA→TMN^) in sleep control, a retrograde lentivirus with Cre-dependent expression of channelrhodopsin-2 (ChR2) was injected into the TMN (Chung et al., 2017). Optogenetic activation of the GABA^POA→TMN^ neurons significantly promoted NREM and REM sleep, while optogenetic inhibition suppressed sleep, demonstrating their sufficient and necessary role in sleep regulation (Figures 1A,B). Optrode recordings allow for the identification of ChR2-expressing neurons to monitor their activity during sleep and wakefulness (Figure 1C). The firing rate of ChR2-tagged GABA^POA→TMN^ neurons was higher during REM and NREM sleep than during wakefulness. Furthermore, gene profiling revealed a variety of molecular markers for the GABA^POA→TMN^ neurons. In particular, cholecystokinin (CCK), corticotropin-releasing hormone (CRH), and tachykinin 1 (TAC1) are highly enriched in GABA^POA→TMN^ neurons, and optogenetic and chemogenetic manipulation demonstrated that these subpopulations of POA neurons are indeed sleep-promoting and necessary for sleep.

Using single-cell RNA sequencing (scRNA-seq) together with multiplexed error robust fluorescence *in situ* hybridization (MERFISH), a recent study revealed that the POA contains a multitude of different cell populations (Moffitt et al., 2018). This approach revealed seven clusters that are enriched in galanin expression, and each cluster was characterized by the expression of distinct marker genes such as peptides, sex steroid, and neuropeptide receptors. About 70% of galanin neurons are GABAergic and a subclass of the inhibitory neurons expresses tyrosine hydroxylase, a marker for neurons involved in maternal care and oxytocin secretion and are located in the sexually dimorphic anteroventral periventricular nucleus (AVPe) in the POA (Scott et al., 2015; Moffitt et al., 2018). This study revealed a staggering heterogeneity of neurons within the POA that awaits to be further characterized using recently developed tools to fully understand how different POA subpopulations generate and coordinate different behaviors.

### Output and Input Pathways of POA Sleep Neurons

Preoptic area of the hypothalamus neurons project to multiple brain regions that are involved in various behaviors (Steininger et al., 2001). In addition to the TMN discussed above, both anterograde and retrograde tracing have shown that POA neurons project to brainstem regions such as the laterodorsal tegmental nucleus (LDT), dorsal raphe (DRN) and median raphe nucleus (MRN), LC, and ventrolateral periaqueductal gray (vlPAG) (Steininger et al., 2001; Lu et al., 2002; Hsieh et al., 2011; Saito et al., 2013; Figure 2). Retrograde tracing from these regions together with c-Fos immunohistochemistry after periods of dark exposure to enhance REM sleep showed retrogradely labeled c-Fos cells in the extended VLPO (Lu et al., 2002), suggesting that POA neurons projecting to LDT, DRN, and LC are involved in REM sleep regulation. Serotonergic DRN and noradrenergic LC cells are silent during REM sleep (Aston-Jones and Bloom, 1981; Heym et al., 1982; Thakkar et al., 1998). The suppression of the DRN and LC during REM sleep is likely caused by GABA release, and one of the potential sources providing GABAergic inputs to these areas is the POA (Luppi et al., 1995; Nitz and Siegel, 1997; Gervasoni et al., 2000; Lu et al., 2002). Increasing the activity of POA neurons through local warming inhibited the DRN cells, providing further evidence for their inhibition by POA neurons (Guzmán-Marín et al., 2000). Thus, these studies suggest that increased activity of POA GABAergic neurons contributes to the suppression of DRN and LC neurons during REM sleep.

Preoptic area of the hypothalamus GABAergic neurons also densely project to the lateral hypothalamus (LH), and monosynaptically restricted rabies tracing confirmed that they innervate hypocretin/orexin (Hcrt/Orx) neurons in the LH (Saito et al., 2013, 2018). Furthermore, whole cell patch clamp recordings of Hcrt/Orx neurons while optogenetically stimulating POA GABAergic axons in the LH showed that the POA axons inhibit Hcrt/Orx neurons through GABA_A_ receptor-mediated synaptic transmission (Saito et al., 2013).

To identify inputs to the VLPO, the retrograde tracer cholera toxin B (CTB) was injected into the VLPO and CTB-labeled neurons were examined throughout the whole brain (Chou et al., 2002). CTB containing neurons were found in multiple brain regions such as the TMN, raphe nuclei, ventrolateral medulla, and LC (Figure 2). Immunohistochemistry confirmed that histaminergic, noradrenergic, and serotonergic fibers densely innervate the POA (Chou et al., 2002) suggesting that monoaminergic nuclei are reciprocally connected with the POA. In addition, the VLPO receives inputs from various hypothalamic regions such as the MnPO, LH, and dorsomedial hypothalamus (DMH). VLPO sleep neurons are thus likely influenced by a variety of brain regions regulating different behaviors and functions.

The impact of monoamines on the activity of putative sleep neurons in the POA was studied *in vitro* using slice physiology. Putative sleep neurons were identified by the presence of low-threshold spikes (LTSs) (Gallopin et al., 2000). Two-thirds of neurons in the VLPO are LTS cells, which matches the proportion of sleep-active neurons *in vivo*. LTS cells are GABAergic and are inhibited by wake-promoting substances such as noradrenaline (NA) and acetylcholine. Some of the LTS cells are either excited or inhibited by serotonin (5-HT), but not histamine (Gallopin et al., 2000, 2005). A recent study using rabies-mediated monosynaptic retrograde tracing showed that POA neurons innervating histaminergic neurons in the TMN and Hcrt/Orx expressing neurons in the LH were potently inhibited by NA and 5-HT (Saito et al., 2018). Thus, putative POA sleep neurons, either defined by the presence of LTS spikes or their connectivity with the TMN histaminergic neurons or LH Hcrt/Orx neurons, receive monoaminergic innervation. Consistent with these findings, electrical stimulation of the LC inhibited the activity in 50% of sleep-active neurons through α_2_-adrenoreceptors (Osaka and Matsumura, 1994, 1995). The inhibited sleep-active neurons were either most active during NREM sleep or during both NREM and REM sleep compared to wakefulness. In contrast, the activity in 47% of wake-active neurons was increased by LC stimulation. These results suggest that sleep and wake-active neurons in the POA receive inhibitory and excitatory inputs from the LC, respectively. Given that electrical stimulation may stimulate different cell types within and surrounding the LC including GABAergic neurons, which have also been implicated in arousal (Breton-Provencher and Sur, 2019), it is important to disentangle the roles of NA- or GABAergic neurons on the brain state through their effects on the POA. Therefore, these results suggest that sleep neurons are inhibited by wake-promoting transmitters such as noradrenaline and acetylcholine, and the reciprocal inhibitory interactions between sleep neurons in the POA and arousal systems may be a key circuit motif underlying the regulation of sleep-wake states (Saper and Fuller, 2017; Scammell et al., 2017).

The POA also receives major inputs from various hypothalamic regions (Figure 2). Histamine neurons in the TMN project to the POA, and histamine indirectly inhibits LTS-type VLPO neurons through local interneurons (Williams et al., 2014). The POA also receives inputs from neurons in the LH; a small fraction of those is positive for melanin-concentrating hormone (MCH) or Hcrt/Orx (21 and 4%, respectively) (Chou et al., 2002). GABAergic neurons in the LH are wake- and REM sleep-active as shown using fiber photometry and directly innervate VLPO neurons that were identified by galanin expression and an inhibitory response to NA (Venner et al., 2019). The DMH has also been shown to innervate the POA (Chou et al., 2002; Chen et al., 2018). In particular, galanin labels a subpopulation of GABAergic neurons in the DMH projecting to the POA (Chen et al., 2018). Optogenetic stimulation of POA-projecting DMH galanin neurons suppressed REM sleep and promoted NREM sleep. Microendoscopy calcium imaging of the POA-projecting galanin neurons in the DMH revealed that their activity is low during REM sleep, and high during NREM sleep. In future studies, it would be of great interest to elucidate which specific POA subregions and cell types the DMH galanin neurons innervate to better understand how the DMH, another key brain region for thermoregulation, interacts with the POA.

### Impact of Somnogens on POA Sleep Neurons

Ishimori (1909) and Legendre and Piéron (1913) discovered that injecting cerebrospinal fluid from a sleep-deprived dog into a normal dog promotes sleep. These studies suggest that prolonged wakefulness leads to the accumulation of sleep-promoting substances, termed somnogens.

Adenosine has been extensively studied as an endogenous somnogen. The adenosine level increases with prolonged wakefulness and decreases throughout sleep (Porkka-Heiskanen et al., 1997). During sleep deprivation, adenosine strongly increases in the basal forebrain and to a lesser extent in the cortex, but not in the other regions that were examined (Porkka-Heiskanen et al., 2000). The site-specific increase of adenosine during sleep deprivation suggests that its sleep-promoting effect is primarily mediated through signaling in the basal forebrain. Adenosine regulates neural activity via two receptors: the inhibitory A1 receptors are widely distributed in the brain while the excitatory A2a receptors are mainly located in the striatum, nucleus accumbens, and olfactory bulb (Porkka-Heiskanen and Kalinchuk, 2011). The impact of adenosine on sleep-active POA neurons was also investigated: Injection of an A2a receptor agonist into the lateral ventricle or subarachnoid space promotes sleep and increases c-Fos expression in GABAergic neurons of the MnPO and VLPO (Scammell et al., 2001; Kumar et al., 2013). Systematic injection of an A2a receptor antagonist instead suppressed sleep deprivation induced expression of c-Fos in MnPO and VLPO neurons (Kumar et al., 2013) and attenuated the activity of the sleep-active neurons during sleep deprivation *in vivo* (Alam et al., 2014).

Prostaglandin D_2_ (PGD_2_) is another powerful sleep-promoting substance. PGD_2_ injection into the subarachnoid space promotes NREM sleep, and injecting it just anterior and ventral to the VLPO induces its largest sleep promoting effect (Matsumura et al., 1994). The sleep promoting effect of PGD_2_ is likely mediated by the leptomeninges, the only area where PGD_2_ receptors are expressed (Urade et al., 1993; Gerashchenko et al., 1998), and the receptor level is highest in the leptomeninges near the VLPO and TMN (Mizoguchi et al., 2001). PGD_2_ injection increased c-Fos expression in the VLPO and this increase was positively correlated with the amount of sleep, indicating that its sleep promoting effect is through leptomeningeal PGD_2_ receptors and subsequent activation of VLPO neurons (Scammell et al., 1998). PGD_2_ infusion to the subarachnoid space near the VLPO/basal forebrain also increased the adenosine level in WT mice, but not in PGD_2_ receptor-deficient mice (Mizoguchi et al., 2001). PGD_2_-induced sleep is reduced by an A2a receptor antagonist suggesting adenosine release mediates the sleep-promoting effect (Satoh et al., 1996; Scammell et al., 2001).

## Role of the POA in Thermoregulation

**FIGURE 3:**
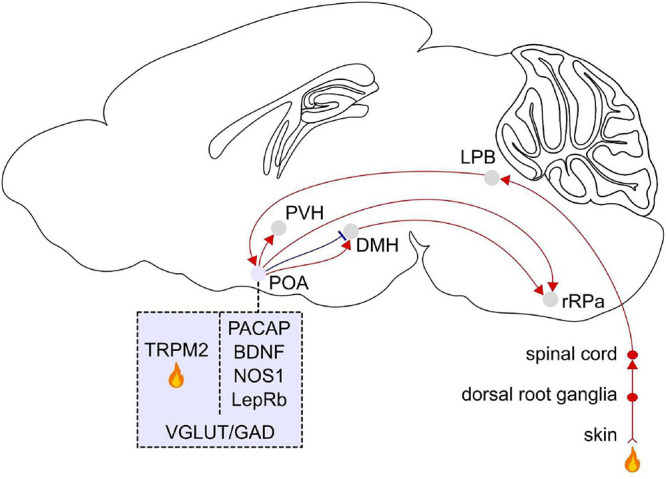
Afferent and efferent projections of POA warm sensitive neurons. Temperature signals are detected in the skin by primary sensory ganglia and then transmitted to the dorsal horn of the spinal cord, the lateral parabrachial nucleus (LPB) in the pons, and the POA. Molecular markers for POA neurons that become activated in response to external warming are pituitary adenylate cyclase-activating polypeptides (PACAP) and brain-derived neurotrophic factor (BDNF), neuronal nitric oxide synthase (NOS1), and the leptin receptor (LepRb). The transient receptor potential M2 (TRPM2) ion channel is a local heat sensor in the POA. Thermoregulatory neurons in the POA project to the dorsomedial hypothalamus (DMH) and rostral raphe pallidus (rRPa). The rRPa is also strongly innervated by the DMH. TRPM2 labeled POA neurons project to the paraventricular hypothalamus (PVH). POA neurons labeled with these markers co-express vesicular glutamate transporter (VGLUT) or glutamic acid decarboxylase (GAD). Sagittal brain scheme adapted from Allen Mouse Brain Atlas (©Allen Brain Atlas API. Available from http://api.brain-map.org).

Since Magoun et al. ’s (1938) seminal discovery that POA warming induces hypothermia in cats, the POA has been demonstrated to be a key brain region integrating peripheral and central temperature information to control the body temperature (Nakamura, 2011; Tan and Knight, 2018). After lesioning or pharmacologically silencing the POA, rats are unable to regulate their core body temperature in response to increases (35–43°C) or decreases (−5–6°C) of the ambient temperature (Lipton, 1968; Carlisle, 1969; Satinoff et al., 1976; Van Zoeren and Stricker, 1976; Osaka, 2004; Ishiwata et al., 2005), demonstrating the necessary role of the POA for autonomous thermoregulation in response to heat or cold exposure. In contrast, POA lesions in rats do not affect behavioral thermoregulatory responses such as warmth seeking or cold seeking (Lipton, 1968; Carlisle, 1969; Satinoff and Rutstein, 1970; Roberts and Martin, 1977; Schulze et al., 1981; Almeida et al., 2006) suggesting that the POA is involved in regulating autonomous, but not behavioral thermoregulation. Warm and cold signals are detected in the skin by primary sensory ganglia and then transmitted to the dorsal horn of the spinal cord, the lateral parabrachial nucleus in the pons and finally relayed to the POA (Figure 3). External heat exposure (31.5–38.5°C) in cats, mice, and rats elevates the activity of neurons in the POA (Nakayama et al., 1961, 1963; Knox et al., 1973; Scammell et al., 1993; Gong et al., 2000; Bachtell et al., 2003; Harikai et al., 2003; Bratincsák and Palkovits, 2004; Yoshida et al., 2005; Tan et al., 2016). In addition, the POA detects the local brain temperature, which increases in response to exercise and fever (Fuller et al., 1998), and local heating of the POA (39–43°C) in cats and rats causes hypothermia and heat dissipation behaviors such as panting and sweating (Magoun et al., 1938; Carlisle, 1966; Carlisle and Laudenslager, 1979). A subset of POA neurons that are activated by local hypothalamic heating also become activated by warming of the skin and spinal cord suggesting that central and peripheral thermal signals converge onto the POA to influence downstream thermoregulatory systems (Wit and Wang, 1968; Boulant and Hardy, 1974). POA neurons that become activated by central or peripheral warming relay temperature information to downstream effectors such as the DMH and rostral raphe pallidus (rRPa), which control peripheral organs to trigger autonomic responses such as suppressing thermogenesis and facilitating heat loss through sweating and vasodilation (Nakamura, 2011; Morrison, 2016; Tan and Knight, 2018; Figure 3). The rRPa is the primary endpoint in the brain that influences thermogenic pathways through the brown adipose tissue (BAT), a heat generating tissue regulating the core body temperature. Activation of the rRPa induces thermogenesis, while inhibition blocks it in rats (Morrison et al., 1999; Nakamura et al., 2002; Morrison, 2003). The rRPa is densely innervated by the DMH, which in turn receives inputs from the POA (Hosoya et al., 1989; Nakamura, 2011). These studies suggest a critical role of the POA in integrating temperature information and triggering behavioral and autonomic responses through their central and peripheral downstream targets to adjust the body temperature.

### Overlapping Circuits for Sleep- and Thermoregulation

Sleep and thermoregulation influence each other. During sleep, central autonomic and thermoregulatory activity is set to low to meet the needs for conserving energy. Sleep neurons in the POA may influence downstream thermoregulatory and central autonomic pathways to maintain this energy saving state, but the exact mechanism is not very well understood. There is also evidence that the thermoregulatory system influences sleep. The amount of sleep is influenced by the ambient temperature and a warm input from the skin and POA warming decreases the latency to sleep. On the other hand, cold exposure reduces and disrupts NREM sleep (Cerri et al., 2005), possibly because the reduced thermogenesis during sleep is not sufficient to maintain a stable core temperature.

The mechanisms for sleep and body temperature regulation anatomically and functionally overlap within the POA. Warming the POA in cats and rats induces relaxation and sleep together with enhanced slow-wave activity in the EEG (Roberts and Robinson, 1969; McGinty et al., 1994). At higher warming levels, they start to pant and stretch to dissipate heat. These studies suggest that POA neurons that become activated upon local warming may provide inputs to sleep promoting neurons to initiate and maintain sleep. The overlap between sleep and thermoregulation circuits was noted early on: the location of electrodes used to locally warm the brain in cats (Roberts and Robinson, 1969) was similar to that of electrodes implanted to promote sleep through electrical stimulation (Sterman and Clemente, 1962a) further suggesting that thermoregulatory processes in the POA may contribute to sleep regulation. To examine whether the sleep and body temperature regulation is mediated by the same POA neurons, single unit recordings combined with local temperature manipulation were performed in the POA of cats and rats. This approach revealed that a subset of POA neurons activated by local warming exhibits an increased discharge rate during NREM sleep compared to wakefulness (Alam et al., 1995, 1996, 1997; Szymusiak et al., 1998). Conversely, a subset of cold-sensitive neurons was more active during wakefulness than during sleep.

Several studies have further investigated the impact of POA lesions on sleep and thermoregulatory behaviors. Cats with POA lesions exhibit a significant decrease in NREM and REM sleep and cannot regulate their body temperature in response to heat (Szymusiak et al., 1991). However, the sleep disturbances were attenuated upon exposure to high ambient temperatures (33°C) that also significantly increased the brain temperature. Similarly in rats, POA lesions decreased sleep and promoted preference to stay in warmer places (30°C) (Ray et al., 2005), which in turn improved sleep. Sleep and body temperature were examined in rats after lesions of the VLPO and ventromedial preoptic area (VMPO) using ibotenic acid (Lu et al., 2000). The VLPO lesion decreased sleep without affecting the body temperature. In contrast, the VMPO lesion did not change sleep, but resulted in a significant increase in the circadian amplitude of body temperature changes. This lesion study suggests that the VMPO and VLPO may be involved in the regulation of thermoregulation and sleep, respectively.

In mice and rats, transitions to sleep are accompanied by a decrease in brain and core body temperature (Obál et al., 1985; Alföldi et al., 1990; Franken et al., 1992a, b; Hoekstra et al., 2019; Sela et al., 2021) suggesting that the brain state drives fluctuations in the body temperature. The brain and cortical temperature during different vigilance states has been further characterized in many mammalian species such as sheep, Djungarian hamsters, and monkeys (Baker and Hayward, 1968; Hayward and Baker, 1968; Deboer et al., 1994; Blessing, 2018). Sustained waking induced by sleep deprivation significantly elevated the cortical temperature in mice suggesting a causal relationship between wakefulness and cortical temperature (Hoekstra et al., 2019). Furthermore, a mathematical model used the sleep-wake sequence to simulate and predict the cortical temperature and found a significant influence of the sleep-wake state on brain temperature (Sela et al., 2021). Sleep fragmentation in mice impairs the reduction in brain temperature during sleep and instead resulted in an increase (Baud et al., 2013). These studies suggest that the transitions between sleep-wake states rapidly change the brain temperature and that a disruption of sleep counteracts the drop in temperature. The circadian rhythm also contributes to changes in body temperature (Wever, 1979; Lavie, 2001). In particular, fluctuations in body and brain temperature have been proposed to follow an ultradian rhythm, termed basic rest-activity cycle (BRAC) (Blessing and Ootsuka, 2016; Blessing, 2018; Goh et al., 2019). Increases in body and brain temperature are shortly followed by BAT thermogenesis in rats and occur approximately every 1–2 h (Ootsuka et al., 2009). The behavioral activity, arterial pressure, heart rate as well as metabolic rate also exhibit an ultradian fluctuations in synchrony with the rest-activity cycle. The role of the POA in controlling the ultradian and circadian regulation of sleep-wake states and temperature is not well understood. POA neurons regulating sleep and/or body temperature may receive circadian signals indirectly from the suprachiasmatic nucleus (SCN) via subparaventricular zone or DMH (Lu et al., 2001; Chou et al., 2002; Deurveilher et al., 2002). However, the sleep-wake state induced changes in brain temperature remained intact in SCN lesioned rats suggesting that the relationship between ultradian changes of body temperature and sleep is independent of circadian clock (Baker et al., 2005). Even in constant darkness, the variations in cortical temperature in rats are mainly determined by changes of sleep-wake states suggesting that the temperature rhythm is not entrained by the light–dark cycle (Franken et al., 1992b). These studies suggest, to a large extent, that the body temperature fluctuations are influenced by sleep-wake states independently of circadian rhythmicity of the SCN. In future studies, it would be interesting to test whether vigilance state-induced body temperature fluctuations may be mediated by the POA sleep-promoting neurons that regulate downstream thermoregulatory pathways.

### Molecular Characterization of POA Thermoregulatory Neurons and Their Pathways

Electrophysiological recordings followed by single cell transcriptomics revealed the expression pattern of neurotransmitters, neuromodulators, receptors, and ion channels in the electrophysiologically identified warm sensitive neurons (WSNs) of mice (Eberwine and Bartfai, 2011). This effort uncovered an unexpected degree of molecular heterogeneity in WSNs, which have been previously thought to form a rather homogeneous population based on electrophysiological recordings and immunohistochemical analysis (Nakayama et al., 1961, 1963; Boulant, 1998). Interestingly, WSNs express various neuropeptides such as galanin, TAC1, somatostatin, and prodynorphin (Eberwine and Bartfai, 2011), and POA neurons expressing these markers have been shown to promote sleep (Xu et al., 2015; Chung et al., 2017; Kroeger et al., 2018; Ma et al., 2019). Furthermore, injecting these neuropeptides into the POA or their axonal projection targets such as the DMH and rRPa induces hyperthermia (Eberwine and Bartfai, 2011), indicating that they are likely secreted in these postsynaptic regions. Single cell transcriptomics further revealed the expression of multiple ion channels and G protein-coupled receptors (GPCRs) including orphan GPCRs (Eberwine and Bartfai, 2011). Given the availability of agonists, antagonists, and modulators for these receptors and channels, their pharmacological roles in the POA can be further investigated to uncover how WSNs integrate various signals to regulate the body temperature.

To identify specific molecular markers for POA neurons controlling the body temperature, phosphoTRAP was employed to profile activated neurons in mice (Knight et al., 2012; Tan et al., 2016). Neural activation leads to the phosphorylation of the ribosomal protein S6, which can be captured to sequence mRNAs. Mice were exposed to warm temperature (37°C), and subsequently activated neurons in the anterior VMPO were sequenced. Gene profiling of these neurons revealed that they specifically express the neuropeptides pituitary adenylate cyclase-activating polypeptide (*Adcyap1*, also known as PACAP) and brain-derived neurotrophic factor (*Bdnf*) (VMPO^PACAP/BDNF^) (Tan et al., 2016). Fiber photometry recordings showed that VMPO^PACAP/BDNF^ neurons are activated in response to warm ambient temperatures (30–42°C) or injection of capsaicin, a transient receptor potential (TRP) V1 agonist, but are non-responsive to cold temperatures, the TRP M8 agonist icilin or several non-thermal stimuli. Whole-cell patch clamp recordings from acute brain slices showed that VMPO^PACAP/BDNF^ neurons are not more thermosensitive than neighboring cells indicating they may not be directly activated by local heat. The peripheral injection of capsaicin rapidly activated VMPO^PACAP/BDNF^ neurons suggesting that TRPV1 sensory fibers may influence their activity. These results suggest that VMPO^PACAP/BDNF^ neurons receive temperature signals from peripheral sensory neurons. Optogenetic stimulation of these neurons promoted hypothermia and induced thermoregulatory behaviors such as nest building. The majority of VMPO^PACAP/BDNF^ neurons are GABAergic, and their major projection target is the DMH (Figure 3). Taken together, VMPO^PACAP/BDNF^ neurons are activated by ambient warmth and coordinate the behavioral and autonomic responses to heat.

Another study discovered the TRPM2 ion channel acts as a heat sensor and thus serves as a marker for WSNs in the POA of mice (Song et al., 2016). TRPM2 is a temperature responsive ion channel expressed in a subpopulation of POA neurons and detects increased temperature in brain slices (38°C) and prostaglandin E2 (PGE_2_)-induced fever *in vivo*. TRPM2 is broadly expressed throughout the POA, whereas PACAP/BDNF expression is localized within the VMPO in mice (Tan et al., 2016). Chemogenetic activation and inhibition of TRPM2-POA neurons decreased and increased the body temperature, respectively. About 30% of TRPM2 neurons are excitatory (Song et al., 2016). Activation of POA excitatory (Vglut2) neurons, but not inhibitory (Vgat) neurons, promoted hypothermia indicating that the hypothermic effect of activating POA TRPM2 neurons are mediated by excitatory signals from POA Vglut2-TRPM2 neurons. One of their projection targets is corticotropin releasing hormone neurons in the paraventricular hypothalamus (PVH), which is part of the hypothalamic-pituitary-adrenal stress response system and also activated by fever (Matsuoka et al., 2003; Figure 3).

The body temperature is tightly interconnected with energy expenditure. Leptin signaling in the brain through its receptor (LepRb) has been shown to mediate energy expenditure, and a subpopulation of POA neurons expressing LepRb (LepRb^POA^ neurons) is involved in thermoregulation of mice (Yu et al., 2016). LepRb^POA^ neurons are activated in response to warm temperatures (30°C), but not upon cold exposure (4°C). Activation of LepRb^POA^ neurons in mice decreased the core body temperature, locomotor activity, and energy expenditure as measured by their oxygen consumption. In addition, stimulation of LepRb^POA^ neurons induced postural extension, a behavioral response to high ambient temperature. The LepRb^POA^ neurons that are responsible for thermoregulation are mainly glutamatergic and send stimulatory signals to the DMH/dorsal hypothalamic area and rRPa in mice (Zhang et al., 2011; Yu et al., 2016; Figure 3).

Previous studies employing c-Fos dependent activity tagging tested whether reactivating neurons that are activated by sleep or warmth promote sleep and/or hypothermia. Capturing POA neurons that are activated during sleep rebound and reactivating them promoted both sleep and hypothermia in mice (Zhang et al., 2015). Another study identified POA neurons that are differentially involved in sleep and/or body cooling (Harding et al., 2018). External warming (32°C) activated two groups of neurons in the MnPO/medial preoptic (MPO) hypothalamic area in mice. The tagged neurons were nitrergic/glutamatergic in the MnPO–MPO and GABAergic in the MPO and their reactivation promoted sleep together with hypothermia or only NREM sleep, respectively. This study demonstrated that the circuits for sleep and thermoregulation overlap, at least partially, in the MnPO–MPO neuronal nitric oxide synthase (NOS1) expressing neurons whereas sleep is instead selectively regulated by MPO-GABAergic neurons.

Galanin neurons in the POA have also been shown to be important for hypothermia associated with NREM sleep in mice (Ma et al., 2019). Chemogenetic activation of galanin neurons induces hypothermia (Kroeger et al., 2018; Ma et al., 2019), suggesting a role in promoting heat loss. Ablating galanin neurons significantly elevated the core body temperature and prevented hypothermia induced by injection of the α2-adrenergic agonist dexmedetomidine (Ma et al., 2019). Surprisingly, fiber photometry recordings showed that Galanin neurons are not activated in response to warm ambient temperatures (25–50°C) (Tan et al., 2016). Given that the galanin population includes both GABAergic and glutamatergic neurons in mice (Moffitt et al., 2018), it is unknown whether GABAergic or glutamatergic galanin neurons or both of these subpopulations regulate the body temperature.

Torpor and hibernation are adaptive behaviors that allow homeothermic animals to reduce their body temperature, metabolic rate, and activity in order to survive in harsh environments with low food supply or cold temperatures (Heller and Ruby, 2004; Geiser, 2013). The laboratory mouse *Mus musculus* is able to undergo short-term shallow torpor by lowering its body temperature and metabolism, resulting in reduced energy consumption (Hudson and Scott, 1979). Two recent studies identified specific circuits in the POA that regulate torpor. Takahashi et al. (2020) found that neurons expressing pyroglutamylated Rfamide peptide (QRFP), in the AVPe and medial preoptic area (MPA) induce a long-lasting hypothermic and hypometabolic state similar to hibernation in mice. The QRFP neurons in the AVPe/MPA are both GABAergic and glutamatergic, and their projections to the DMH mediate the hibernation-like state. Activation of QRFP neurons decreases the body temperature and oxygen consumption, while keeping the metabolism at a low rate. No tissue damage was found following the hibernation-like state, and mice spontaneously recovered without external stimulation. Furthermore, activation of calcium/calmodulin-dependent protein kinase II expressing neurons including QRFP neurons in the AVPe/MPA (Takahashi et al., 2020) and injection of the glutamatergic agonist *N*-methyl-D-aspartic acid (NMDA) into the MPA or VLPO (Conceição et al., 2019) also induced a hypometabolic state in rats, a species that does not enter hibernation and daily torpor, indicating that rats share with mice circuits underlying the regulation of hypothermia and hypometabolism. QRFP has also been implicated in regulating food intake, sympathetic regulation, and anxiety and is expressed in other hypothalamic regions (Takayasu et al., 2006; Okamoto et al., 2016). Hrvatin et al. (2020) identified a population of *Adcyap1*-positive cells in the anterior and ventral portions of the medial and lateral preoptic area (avMLPA^Adcyap1^), which control fasting-induced torpor in mice. Activating avMLPA^Adcyap1^ neurons decreased the body temperature and motor activity whereas inhibition disrupted torpor induction, maintenance, and arousal. Fiber photometry showed that their activity increased in response to cold exposure (10°C) and changed during the torpor-like state; their baseline level decreased, while the frequency of Ca^2+^-dependent events increased. Inducing a torpor-like state in non-torpid species such as human patients experiencing trauma from cardiac arrest, organ transplantation, major cardiac, and brain surgery could provide a tool to reduce organ damage (Aslami and Juffermans, 2010; Bouma et al., 2012). Central pharmacological activation of the A1 adenosine receptor was shown to induce hypothermic, torpor-like states in rats when exposed to cold ambient temperatures (15°C) suggesting the potential therapeutic role of targeting this receptor to induce a hypothermic state in humans (Tupone et al., 2013, 2016).

Fever, a significant body temperature increase in response to infections and inflammation is an evolutionarily conserved phenomenon and necessary to help the immune system fight infections and inhibit the growth of microorganisms (Blomqvist and Engblom, 2018). During this state, the presence of bacteria or viruses is sensed by immune cells in the blood leading to the production of cytokines and prostaglandins (Evans et al., 2015). The POA is also activated by fever accompanying an enhanced immune response. Injection of lipopolysaccharide (LPS), common bacterial cell walls, triggers immune responses including fever and increases the number of c-Fos-labeled neurons in the POA in rats, especially within the ventromedial part, indicating a role in initiating the fever response (Elmquist et al., 1996). LPS-induced fever is absent in mice deficient in microsomal prostaglandin E synthase-1 (mPGES-1), which lack PGE_2_ synthesis, suggesting that fever is mediated by PGE_2_ (Engblom et al., 2003). Microinjection of PGE_2_ into the VMPO induces fever and c-Fos expression in rats, demonstrating that the VMPO is an important site through which PGE_2_ promotes fever (Scammell et al., 1996). In particular, PGE_2_ has been shown to act on warm-sensitive thermoregulatory neurons in the hypothalamus (Saper and Breder, 1994; Saper et al., 2012). PGE_2_ activates one of its receptors, EP3R, expressed in excitatory VMPO/MnPO neurons to mediate the fever response in mice (Machado et al., 2020). LPS-induced fever is also mediated by PGE_2_ action through the EP3R receptors in VMPO/MnPO glutamatergic neurons and is eliminated when these neurons are ablated. Glutamatergic EP3R-expressing POA neurons directly project to the rRPa and this pathway may mediate inflammatory fever (Yoshida et al., 2009; Machado et al., 2020). Taken together, these studies on torpor, energy homeostasis, and fever revealed a variety of molecular markers that specifically label thermoregulatory neurons in the POA.

## Discussion

The POA comprises many subdivisions, and the recent identification of novel molecular markers that specifically label neurons regulating sleep or body temperature helped to precisely define the subregions and cell types controlling these fundamental behaviors. Furthermore, newly developed tools greatly improved the temporal and spatial resolution for recording and manipulating these neurons providing important insights into the underlying circuit mechanisms (Figure 1).

However, the extent of anatomical overlap between warm-sensitive and sleep-promoting POA neurons is still largely unknown. Consequently, the projection pattern of such neurons and their interaction with other regions involved in sleep and temperature control is still unknown. An interesting hypothesis to test in future studies is whether neurons involved in both behaviors send axonal projections to both sleep and temperature-regulatory downstream areas. The location of newly identified sleep and thermoregulatory neurons partially overlap. Previous studies indicated that a subset of POA neurons activated by local warming becomes activated during NREM sleep (Alam et al., 1995, 1996, 1997; Szymusiak et al., 1998). It is unknown whether the coordination of sleep and thermoregulation is mediated by neurons that regulate both behaviors, or whether it is the result of local or long-range interactions between separate sets of POA sleep and thermoregulatory neurons. Recent studies provided a list of molecular markers that label sleep and/or thermoregulatory neurons in the POA of mice (Figures 2, 3). Systematic investigation of their anatomical overlap, local synaptic interactions and activity during sleep and in response to local and external temperature changes will help to understand how sleep and thermoregulation are orchestrated at the circuit level. Another possible circuit motif underlying the coordination of both behaviors may be common postsynaptic targets. Given that the body temperature rapidly drops at the onset of NREM sleep, a subset of NREM-active cells may project to areas involved in temperature regulation such as the medullary sympathetic premotor neurons that regulate body temperature and peripheral vasculature (Nakamura, 2011). Elucidating the extent to which the sleep-regulatory neurons target critical nodes of the thermoregulatory pathway such as the DMH and rRPa or the warm-sensitive neurons may in turn innervate regions controlling sleep will greatly enhance our understanding of the coordinated regulation of sleep and body temperature. Furthermore, given that the majority of novel molecular markers has been identified in mice for which most recent techniques are available and the thermoregulatory and sleep behaviors vary across species, it is important to elucidate the circuits regulating these behaviors also in other animals than mice and to identify conserved mechanisms.

Preoptic area of the hypothalamus sleep and thermoregulatory neurons express a variety of peptidergic markers as well as receptors and channels (Eberwine and Bartfai, 2011; Moffitt et al., 2018). An important area of future research is to investigate the functional role of these peptides and the signaling pathways initiated by these receptors/channels using recently developed tools such as sensors to detect the release of neurotransmitters and modulators or CRISPR/Cas9 gene editing to interrogate gene function within molecularly-defined neurons (Yamaguchi et al., 2018; Jing et al., 2019; Sabatini and Tian, 2020).

In addition to thermoregulation, sleep is also closely interconnected with metabolism. During NREM sleep, the energy metabolism is strongly reduced. Chronic sleep fragmentation in mice prevents the reduction of the brain temperature during sleep and leads to increased food intake and glucose intolerance suggesting that poor-quality sleep may contribute to development of metabolic dysfunctions (Baud et al., 2013). Similarly, laboratory and epidemiologic studies suggest that poor sleep might promote the development of metabolic disorders (Spiegel et al., 2009). Local warming of the POA reduces feeding and POA lesions disrupt feeding in response to changes in the ambient temperature, suggesting an overlap of the mechanisms regulating metabolism and thermoregulation within the POA (Andersson and Larsson, 1961; Hamilton and Brobeck, 1966). LepRb expressing neurons in the POA become activated after exposure to warm and neutral ambient temperatures (22–30°C) and modulate feeding and body weight by controlling BAT thermogenesis demonstrating their role in energy homeostasis and body weight regulation in a temperature-dependent manner (Yu et al., 2016). Infusion of glucose to the POA promotes NREM sleep by increasing the activity of sleep-promoting neurons further suggesting the link between sleep and metabolism (Varin et al., 2015). The POA sleep- and/or thermo-regulatory neurons may project to downstream circuits involved in feeding and energy expenditure such as LH to regulate the overall energy metabolism. In addition to sleep, energy metabolism is also influenced by the circadian rhythm. For example, mice with targeted disruption of the core clock gene *Clock* develop obesity and metabolic syndromes suggesting that this circadian clock gene plays an important role in energy balance (Turek et al., 2005). For future studies, it would be interesting to test to what extent circadian influences on the metabolism are mediated by indirect inputs from the SCN to the POA sleep neurons (Lu et al., 2001; Chou et al., 2002; Deurveilher et al., 2002).

Different types of sleep disorders, especially those associated with aging, may be influenced by altered body temperature rhythms or dysregulation of vasodilation resulting in an impairment to prepare the body for sleep (Foley et al., 1995; Ozaki et al., 1996; Pache et al., 2001; Watanabe et al., 2003; Fronczek et al., 2006; Kräuchi, 2007; Lack et al., 2008; Raymann and Van Someren, 2008). Delayed temperature rhythms or dysfunctions in sensing and transferring temperature information to the neurons controlling sleep within the POA may contribute to attenuate the sleep onset in the elderly. Elucidating how POA neurons receiving peripheral temperature signals interact with the sleep-promoting neurons and integrate circadian rhythms, and how these circuits become dysfunctional with aging will greatly improve our current therapeutics for sleep disorders.

## Author Contributions

Both authors wrote the manuscript, made the figures, contributed to the article and approved the submitted version.

## Conflict of Interest

The authors declare that the research was conducted in the absence of any commercial or financial relationships that could be construed as a potential conflict of interest.
